# Effects of meropenem plus ulinastatin on inflammatory response and complications in patients with severe septic shock

**DOI:** 10.3389/fmed.2026.1860367

**Published:** 2026-07-21

**Authors:** Dan Wu, Wei Ni, Xinying Zhang, Xiaodong Liu

**Affiliations:** Department of Emergency Medicine, Nanjing First Hospital, Nanjing Medical University, Nanjing, China

**Keywords:** clinical outcomes, coagulation function, inflammatory response, lactate clearance, meropenem, severe septic shock, ulinastatin

## Abstract

**Aim:**

To evaluate whether receipt of ulinastatin in addition to meropenem-based standard care was associated with inflammatory, coagulation, perfusion, and clinical outcomes in patients with severe septic shock.

**Methods:**

This single-center retrospective, non-randomized comparative cohort study included 106 consecutive patients with severe septic shock treated between January 2023 and November 2025. Patients were classified according to treatment received in routine clinical practice: meropenem-based standard care plus ulinastatin (observation group, *n* = 53) or meropenem-based standard care alone (control group, *n* = 53). Twenty-eight-day all-cause mortality was presented as the principal objective patient-centered clinical outcome, whereas the total effective rate was reported only as an exploratory secondary outcome.

**Results:**

The overall response rate in the observation group presented higher at 90.6% (48/53) versus 73.6% (39/53) in the control group (*p* < 0.05). Post-treatment, the observation group presented lower levels of CRP, PCT, IL-6, IL-8, along with TNF-α (*p* < 0.05). For coagulation, post-treatment Fib levels were lower in the observation group compared to the control group, while PT and APTT were markedly shortened in the observation group (*p* < 0.05). Hemodynamic parameters—heart rate (HR), mean arterial pressure (MAP), along with cardiac index (CI)—showed greater improvement in the observation group (*p* < 0.05). Regarding immunity, the observation group presented higher CD4^+^ T cell counts and CD4^+^/CD8^+^ ratios, as well as significantly lower CD8^+^ T cell counts versus the control group (*p* < 0.05). The 6-h and 24-h lactate clearance rates were significantly higher in the observation group (*p* < 0.001 for both). ICU stay and mechanical ventilation duration were shorter in the observation group (*p* < 0.001 for both). The complication rate was 9.4% (5/53) in the observation group, which was lower than the 24.5% (13/53) seen in the control group (*p* < 0.05).

**Conclusion:**

In this single-center retrospective cohort, receipt of ulinastatin in addition to meropenem-based standard care was associated with more favorable inflammatory, coagulation, lactate-clearance, hemodynamic, immune, and resource-use outcomes. Because treatment was not randomized and residual confounding cannot be excluded, these findings should not be interpreted as proof of causality or synergy.

## Introduction

Septic shock represents a serious complication of sepsis, defined by sustained hypotension that remains unresolved after adequate fluid resuscitation (1). This life-threatening condition is linked to high morbidity and mortality, greatly endangering patient outcomes. Data from the Global Burden of Disease Study indicate a continuing increase in sepsis incidence, with around 48.9 million cases and 11 million sepsis-attributable deaths occurring globally each year (2). Severe septic shock (SSS) constitutes a considerable portion of these cases, with fatality rates ranging between 30 and 50% (3).

The pathophysiology of septic shock involves a complicated interplay of excessive inflammatory response, immune dysfunction, and microcirculatory disturbances. When pathogenic microorganisms enter the bloodstream, they can induce a systemic inflammatory response syndrome (SIRS), featured by the excessive release of pro-inflammatory cytokines (4). This uncontrolled inflammatory cascade results in endothelial injury, heightened vascular permeability, vasodilation, and eventually multiple organ dysfunction syndrome (MODS) (5). Additionally, the inflammatory response triggers the coagulation system, leading to disseminated intravascular coagulation (DIC), which is featured by the depletion of coagulation factors, prolongation of prothrombin time (PT) and activated partial thromboplastin time (APTT), along with diminished fibrinogen (Fib) levels. These changes further promote microvascular thrombosis and worsen organ dysfunction (6). Lactate elevation is a hallmark of tissue hypoperfusion in septic shock, and lactate clearance rate has emerged as an important prognostic marker, with higher clearance rates associated with improved survival (7). Therefore, effective management of septic shock requires not only prompt and appropriate antimicrobial therapy but also modulation of the excessive inflammatory response, restoration of coagulation homeostasis, improvement of tissue perfusion, and preservation of immune function.

Meropenem belongs to a broad-spectrum carbapenem antibiotic with powerful activity against most Gram-positive and Gram-negative bacteria, anaerobes included. It is extensively applied as first-line empirical therapy for severe infections, including sepsis and septic shock, due to its excellent tissue penetration, favorable safety profile, and low potential for cross-resistance (8). According to the 2016 Surviving Sepsis Campaign guidelines, broad-spectrum antibiotics—such as carbapenems—should be initiated within the first hour after septic shock is identified (9). However, antimicrobial therapy alone may not adequately control the hyperinflammatory state, and persistent excessive inflammation contributes to ongoing tissue damage and poor outcomes.

Ulinastatin is a urinary trypsin inhibitor isolated from human urine that possesses broad-spectrum protease inhibitory activity. It inhibits the activity of various proteases such as trypsin, chymotrypsin, elastase, and hyaluronidase, thereby stabilizing lysosomal membranes, reducing the release of inflammatory mediators, as well as alleviating tissue damage (10). Numerous studies have demonstrated that ulinastatin can attenuate the inflammatory response, improve organ function, and decrease death rate in patients suffered from sepsis and septic shock (11). Additionally, ulinastatin has been shown to exert protective function on the coagulation system by inhibiting excessive activation of the coagulation cascade and preserving endothelial function (12). Furthermore, ulinastatin may improve microcirculatory perfusion and enhance lactate clearance by reducing endothelial dysfunction and stabilizing hemodynamics (13). By inhibiting the excessive activation of inflammatory cascades, ulinastatin may complement the antimicrobial effects of meropenem, potentially leading to improved clinical outcomes.

The combination of meropenem and ulinastatin addresses both antimicrobial treatment and modulation of the host inflammatory response. However, evidence regarding the comparative outcomes of adding ulinastatin to meropenem-based care in severe septic shock remains limited. Therefore, this study evaluated associations between receipt of adjunctive ulinastatin and inflammatory response, coagulation function, lactate clearance, and clinical outcomes.

## Methods

### Study subjects

This was a single-center retrospective, non-randomized comparative cohort study of 106 consecutive patients with severe septic shock treated at Nanjing First Hospital from January 2023 to November 2025. Eligible patients were identified from the hospital records and classified according to the treatment actually received in routine clinical practice: meropenem-based standard care plus ulinastatin (observation group, *n* = 53) or meropenem-based standard care alone (control group, *n* = 53). Treatment assignment was determined by the treating clinician.

Inclusion criteria: (1) Diagnosis of SSS meeting Sepsis-3 criteria; (2) Age between 18 and 80 years; (3) Time from onset to admission ≤24 h; (4) Signed informed consent from patients or their legal representatives.

Exclusion criteria: (1) Pregnant or lactating women; (2) Known allergy to meropenem, ulinastatin, or any carbapenem antibiotics; (3) Severe underlying diseases with life expectancy <6 months; (4) Immunosuppressive therapy or history of organ transplantation; (5) Participation in other clinical trials within the previous 3 months; (6) Incomplete clinical data; (7) Preexisting coagulation disorders or anticoagulant therapy.

The study was approved by the Institutional Ethics Review Board of Nanjing First Hospital, Nanjing Medical University (Approval No.: 2023NJ0057_L). All patient data were collected during the approval validity period (January 2023–November 2025). Written informed consent was obtained from the patients or their legal representatives, as stated in the eligibility criteria. The study was conducted in accordance with the Declaration of Helsinki.

### Treatment methods

In line with the Surviving Sepsis Campaign guidelines, all patients underwent standard symptomatic management, which comprised early goal-directed fluid resuscitation, vasopressor support using norepinephrine, mechanical ventilation when indicated, and continuous renal replacement therapy (CRRT) for those with acute kidney injury.

Control group: Patients received intravenous meropenem (Meropenem for Injection, Manufacturer: Hanhui Pharmaceutical Co., Ltd., Batch number: 25123511, specification: 0.5 g/vial) at a dose of 1 g (2 vials) every 8 h, with dose adjustment according to renal function.

Observation group: In addition to standard symptomatic management and meropenem, patients received intravenous ulinastatin (Ulinastatin for Injection; Techpool Bio-Pharma Co., Ltd.; batch no. 032509054; 100,000 units/vial) at a dose of 200,000 units diluted in 100 mL normal saline and infused over 30 min three times daily for 7 consecutive days.

Ulinastatin was administered for 7 consecutive days. Patients were followed during a 14-day treatment and observation period; however, this period did not represent a fixed 14-day course of all medications. Meropenem and other supportive treatments were continued, adjusted, or discontinued according to each patient’s clinical condition, renal function, ICU discharge, or death. Inflammatory, coagulation, hemodynamic, and immune-function indicators were assessed on day 7 because this time point coincided with completion of the planned 7-day ulinastatin course and was used to evaluate the short-term biological response at the end of adjunctive treatment. The exploratory clinical response was assessed at day 14, whereas all-cause mortality was assessed through day 28.

### Observation indicators

The 28-day all-cause mortality was treated as the principal objective patient-centered clinical outcome and was analyzed separately. The total effective rate was retained only as an exploratory secondary outcome. At day 14, clinical response was retrospectively determined from the medical records according to changes in clinical manifestations, hemodynamic stability, vasopressor requirements, and the occurrence of new organ dysfunction. Two attending physicians independently reviewed the medical records, and disagreements were resolved through consultation with a senior intensivist. The assessors were aware of the treatment received because treatment information was documented in the medical records. At day 14, response was categorized from the medical record as follows: (1) markedly effective: clinical manifestations and signs resolved, hemodynamic parameters returned to normal, vasopressor support was discontinued, and no new organ dysfunction occurred; (2) effective: clinical manifestations and signs improved and hemodynamic parameters stabilized with a reduced vasopressor requirement; and (3) ineffective: manifestations did not improve or worsened, or death occurred. Total effective rate = (markedly effective + effective)/total number of patients ×100%.

Inflammatory response and coagulation function indicators: Fasting venous blood (3 mL) was gathered from patients before treatment and on day 7 post-treatment. Samples were centrifuged, and serum was separated. Levels of C-reactive protein (CRP) were measured by immunoturbidimetry; procalcitonin (PCT) was measured by chemiluminescence immunoassay; IL-6, IL-8, along with TNF-α were examined by enzyme-linked immunosorbent assay (ELISA). Levels of Fib, PT, along with APTT were measured utilizing a coagulation analyzer by the clotting method.

Lactate clearance rate: Arterial blood samples were gathered at admission (baseline) and at 6 h and 24 h after initiation of treatment. Blood lactate levels were measured by means of a blood gas analyzer. Lactate clearance rate was calculated as (baseline lactate level – lactate level at specified time point) / baseline lactate level × 100%.

Hemodynamic parameters: Heart rate (HR), mean arterial pressure (MAP), along with cardiac index (CI) were monitored continuously using a bedside monitor (Philips IntelliVue MX800). Measurements were recorded before treatment and on day 7 post-treatment.

Immune function indicators: Peripheral blood samples were obtained before treatment and on day 7 post-treatment. CD4^+^ and CD8^+^ T lymphocyte counts were assessed by flow cytometry (BD FACSCalibur, BD Biosciences, United States) using monoclonal antibodies. The CD4^+^/CD8^+^ ratio was subsequently calculated.

Clinical outcomes: ICU length of stay (days) and duration of mechanical ventilation (days) were recorded for all patients.

Safety and clinical events: ARDS, AKI, DIC, and MODS occurring during hospitalization were recorded as non-fatal complications, while all-cause mortality within 28 days was analyzed separately and was not classified as a complication. Individual patients could experience more than one non-fatal event; therefore, event-specific counts were non-mutually exclusive. The composite ‘any non-fatal complication’ represented the number of patients with at least one such event, with each patient counted once.

### Statistical analysis

SPSS version 26.0 was used. The distribution of continuous variables was assessed using histograms, Q–Q plots, and the Shapiro–Wilk test. Homogeneity of variance was assessed using Levene’s test. Approximately normally distributed variables with homogeneous variances were summarized as mean ± standard deviation and compared using independent-samples *t*-tests for between-group comparisons or paired *t*-tests for within-group comparisons. When the homogeneity-of-variance assumption was not met, Welch’s *t*-test was used. Non-normally distributed variables were summarized as median (interquartile range) and compared using the Mann–Whitney U test or Wilcoxon signed-rank test, as appropriate.

Effect estimates with 95% confidence intervals were reported. To reduce the risk of inflated type-I error due to multiple comparisons, the Benjamini-Hochberg false discovery rate procedure was applied within each family of related secondary outcomes. For continuous outcomes, between-group mean differences with 95% confidence intervals were reported; for categorical outcomes, unadjusted risk ratios with 95% confidence intervals were calculated. Statistical significance was defined as two-sided *p* < 0.05.

## Results

### Baseline characteristics

No discrepancies were found between both groups regarding age, sex, body mass index (BMI), APACHE II score, SOFA score, baseline lactate level, or site of infection (*p* > 0.05) (Table 1).

**Table 1:** Baseline characteristics of patients in both groups.

Item	Observation group (*n* = 53)	Control group (*n* = 53)	*t*/χ^2^	*P*
Age (years)	57.12 ± 9.03	56.34 ± 8.67	0.452	0.652
Sex			0.152	0.697
Male	32 (60.4)	30 (56.6)		
Female	21 (39.6)	23 (43.4)		
BMI (kg/m^2^)	23.45 ± 2.34	23.12 ± 2.45	0.704	0.483
APACHE II score	22.34 ± 4.56	21.98 ± 4.32	0.420	0.675
SOFA score	10.23 ± 2.34	9.98 ± 2.21	0.565	0.573
Infection site			0.182	0.913
Pulmonary infection	28 (52.8)	26 (49.1)		
Abdominal infection	15 (28.3)	16 (30.2)		
Other	10 (18.9)	11 (20.7)		

### Clinical efficacy

At day 14, the exploratory total effective rate was 90.6% (48/53) in the observation group and 73.6% (39/53) in the control group (risk ratio, 1.23; 95% CI, 1.02–1.48; *p* = 0.022) (Table 2).

**Table 2:** Exploratory clinical response at day 14.

Group	*n*	Markedly effective, *n* (%)	Effective, *n* (%)	Ineffective, *n* (%)	Total effective rate, *n* (%)	Risk ratio (95% CI)	*P*
Observation group	53	32 (60.4)	16 (30.2)	5 (9.4)	48 (90.6)	1.23 (1.02–1.48)	0.022
Control group	53	25 (47.2)	14 (26.4)	14 (26.4)	39 (73.6)	Reference	–

### Inflammatory response indicators

**Figure 1:**
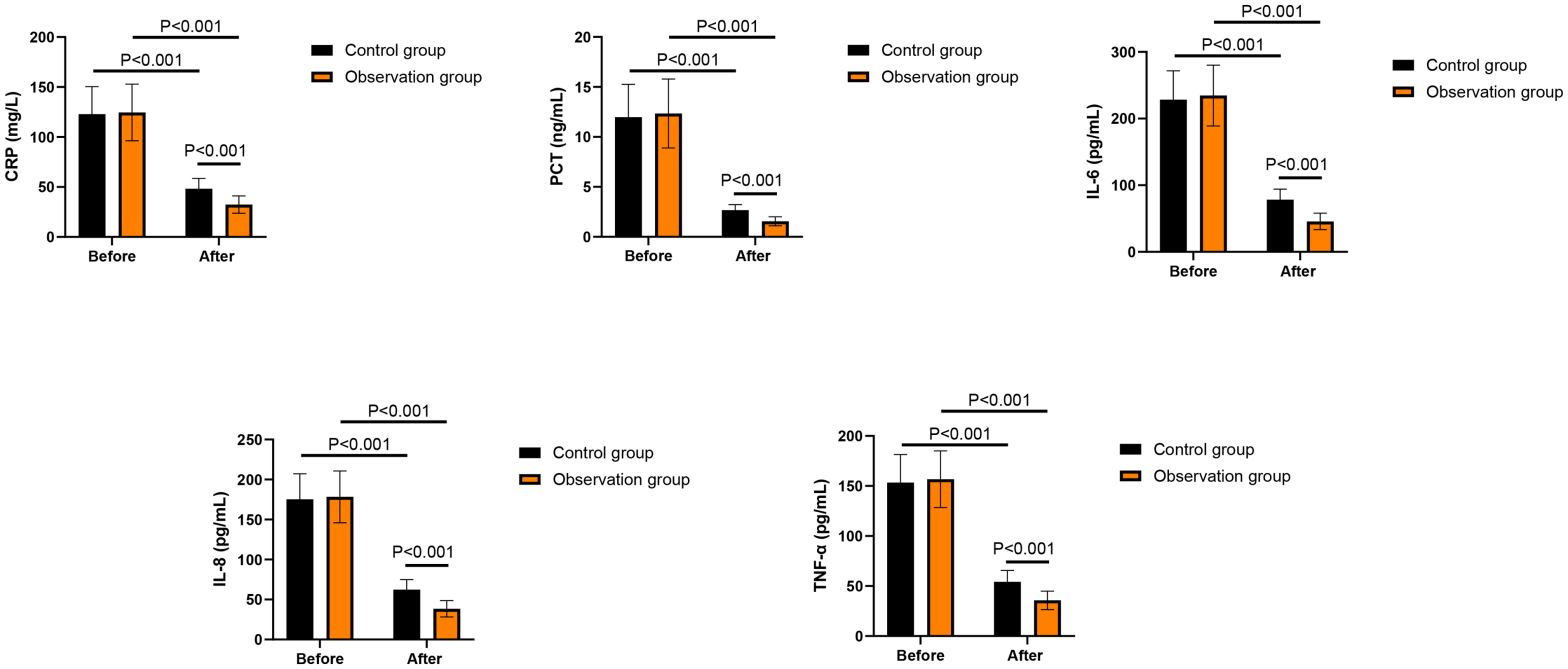
Inflammatory-response indicators before and after treatment in the two groups. Data are presented as mean ± standard deviation.

At baseline, no statistically significant between-group differences were observed in CRP, PCT, IL-6, IL-8, or TNF-alpha levels (all *p* > 0.05). After treatment, all five inflammatory markers were lower in the observation group than in the control group. The between-group mean differences were −15.78 for CRP (95% CI, −19.46 to −12.10), −1.11 for PCT (95% CI, −1.31 to −0.91), −32.78 for IL-6 (95% CI, −38.22 to −27.34), −23.89 for IL-8 (95% CI, −28.30 to −19.48), and −18.56 for TNF-alpha (95% CI, −22.54 to −14.58). All comparisons remained statistically significant after Benjamini-Hochberg false discovery rate correction (all adjusted *p* < 0.001; Figure 1; Supplementary Table S1).

### Coagulation function indicators

**Figure 2:**
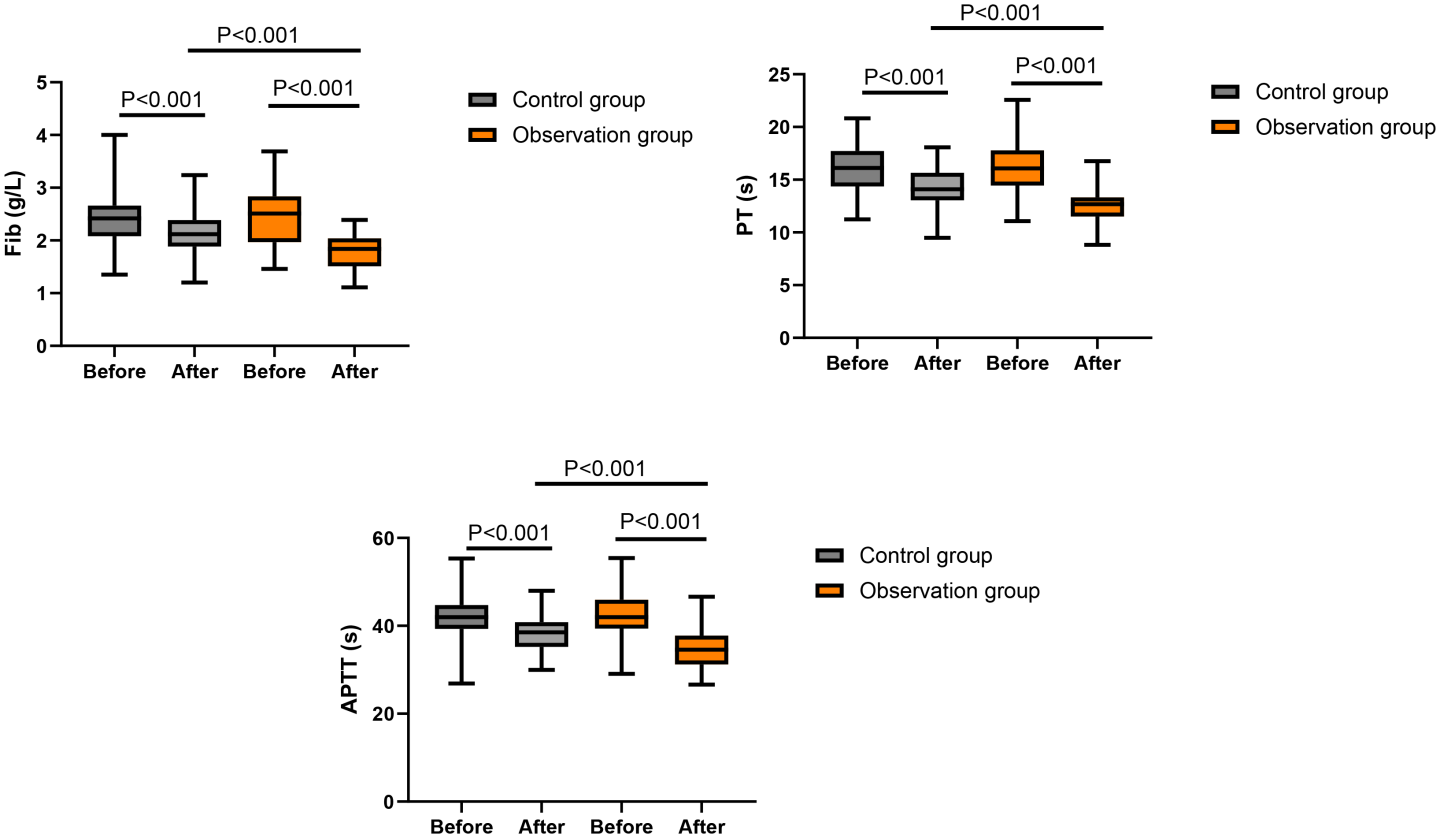
Coagulation-function indicators before and after treatment in the two groups. Data are presented as mean ± standard deviation.

At baseline, no statistically significant between-group differences were observed in Fib, PT, or APTT levels (all *p* > 0.05). After treatment, Fib levels were lower in the observation group than in the control group (1.78 ± 0.34 vs. 2.12 ± 0.42 g/L; mean difference, −0.33 g/L; 95% CI, −0.48 to −0.18). PT was shorter in the observation group than in the control group (12.45 ± 1.56 vs. 14.23 ± 1.78 s; mean difference, −1.78 s; 95% CI, −2.42 to −1.14). APTT was also shorter in the observation group than in the control group (34.56 ± 4.23 vs. 38.45 ± 4.56 s; mean difference, −3.89 s; 95% CI, −5.59 to −2.19). All comparisons remained statistically significant after Benjamini-Hochberg false discovery rate correction (all adjusted *p* < 0.001; Figure 2; Supplementary Table S1).

### Lactate clearance rates

**Figure 3:**
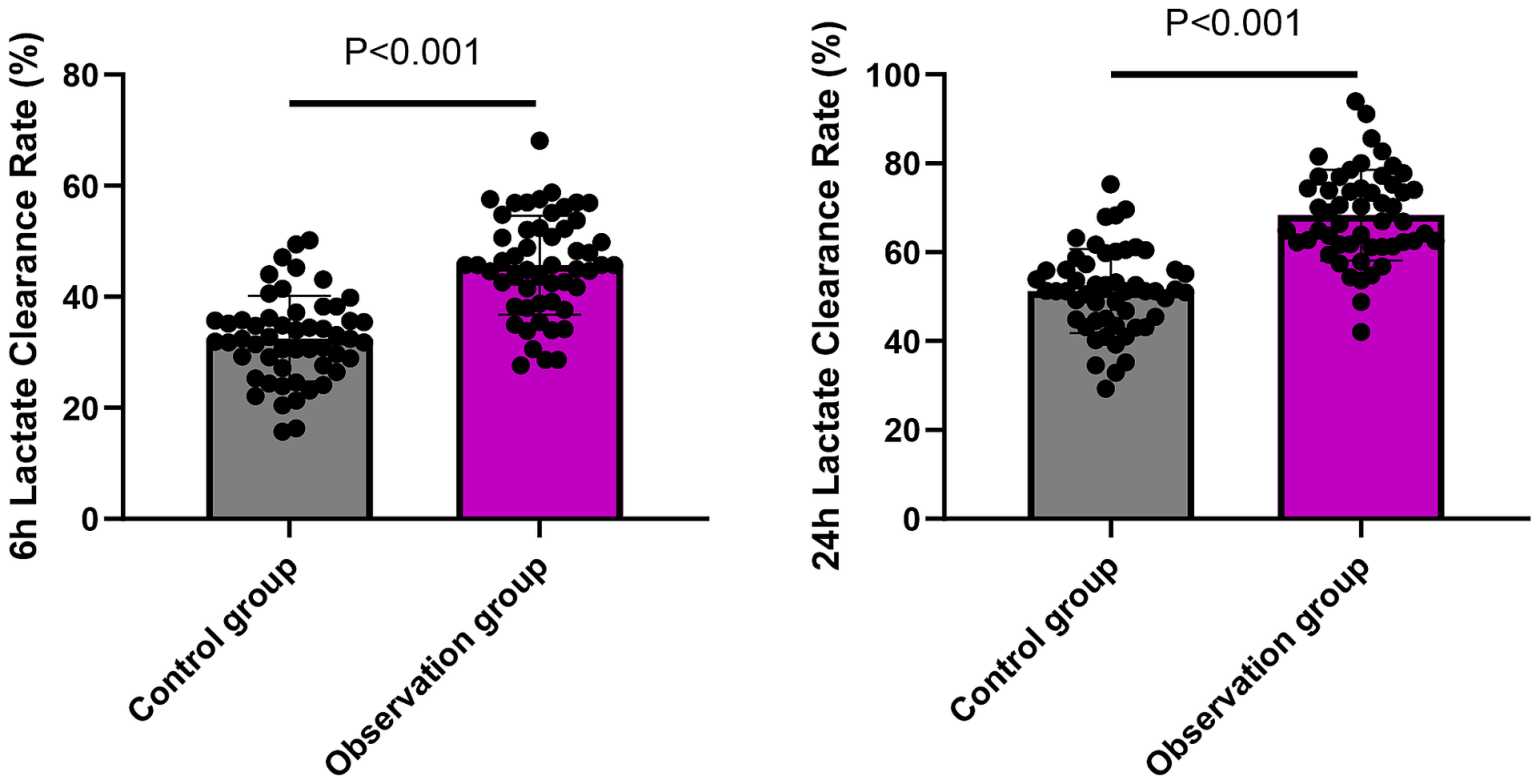
Lactate clearance rates at 6 and 24 h in the two groups. Data are presented as mean ± standard deviation with individual patient values. Between-group comparisons were performed using independent-samples *t*-tests.

The lactate-clearance data at both 6 and 24 h were approximately normally distributed in each group, as assessed using the Shapiro–Wilk test. At 6 h, the mean lactate clearance rate was significantly higher in the observation group than in the control group (45.67 ± 8.92% vs. 32.45 ± 7.68%; mean difference, 13.22 percentage points; 95% CI, 10.01 to 16.43; *t* = 8.172; *p* < 0.001). At 24 h, the mean lactate clearance rate was also significantly higher in the observation group than in the control group (68.34 ± 10.23% vs. 51.23 ± 9.45%; mean difference, 17.10 percentage points; 95% CI, 13.31 to 20.89; *t* = 8.939; *p* < 0.001) (Figure 3; Supplementary Table S1).

### Hemodynamic parameters

**Figure 4:**
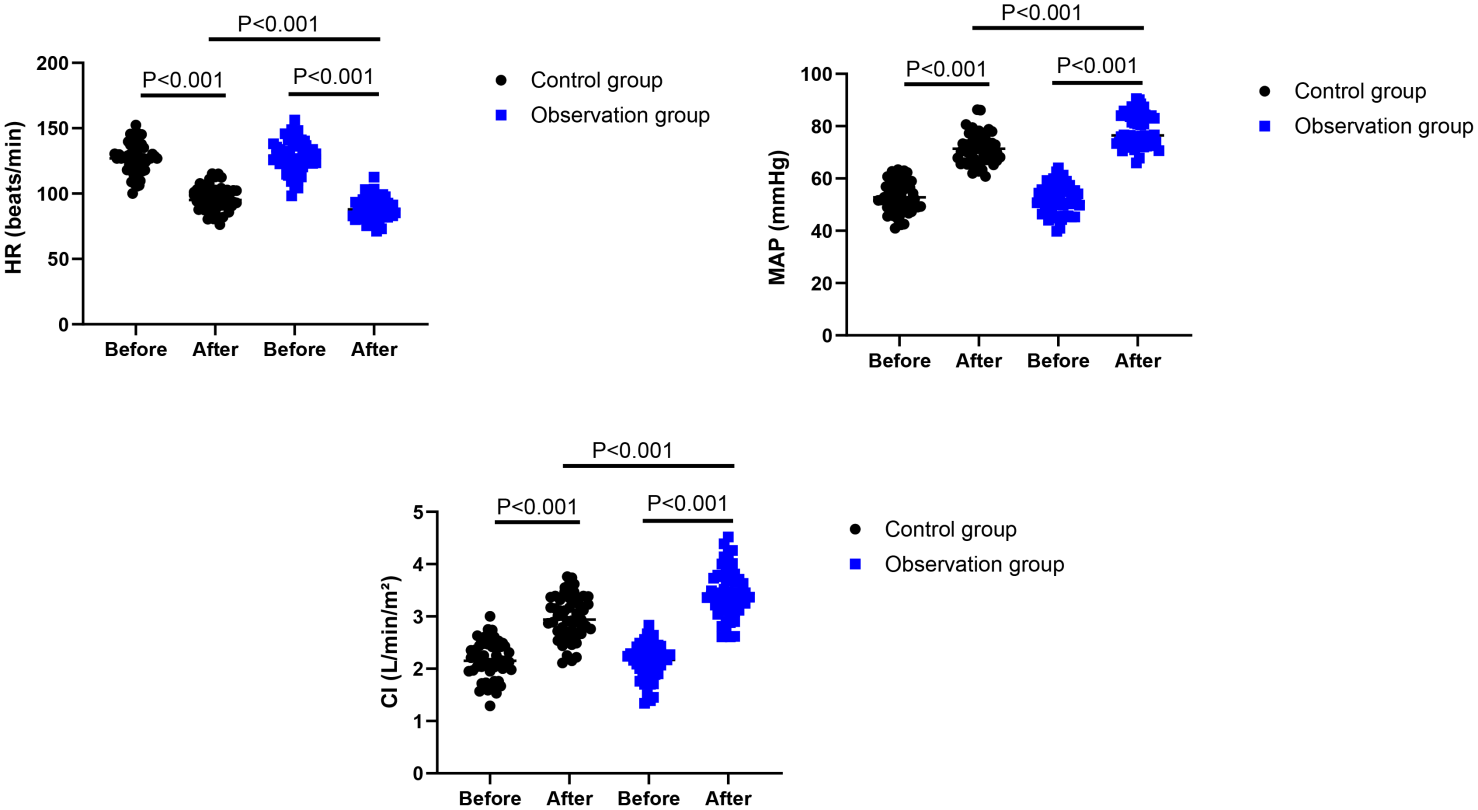
Hemodynamic parameters before and after treatment in the two groups. HR and CI are presented as mean ± standard deviation and were compared using independent-samples *t*-tests. MAP is presented as median (interquartile range) and was compared using the Mann–Whitney U test.

At baseline, no statistically significant between-group differences were observed in HR, MAP, or CI (all *p* > 0.05). After treatment, HR was significantly lower in the observation group than in the control group (88.34 ± 8.45 vs. 96.23 ± 9.12 bpm; mean difference, −7.88 bpm; 95% CI, −11.27 to −4.49). Cardiac index was significantly higher in the observation group (3.45 ± 0.45 vs. 2.98 ± 0.41 L/min/m^2^; mean difference, 0.48 L/min/m^2^; 95% CI, 0.31 to 0.65). Because post-treatment MAP was not normally distributed in the observation group, MAP was summarized as median (interquartile range) and compared using the Mann–Whitney U test. Median MAP was higher in the observation group than in the control group [76.49 (73.37–83.73) vs. 71.34 (67.11–74.54) mmHg; *p* < 0.001]. All three comparisons remained statistically significant after Benjamini-Hochberg false discovery rate correction (all adjusted *p* < 0.001; Figure 4; Supplementary Table S1).

### Immune function indicators

**Figure 5:**
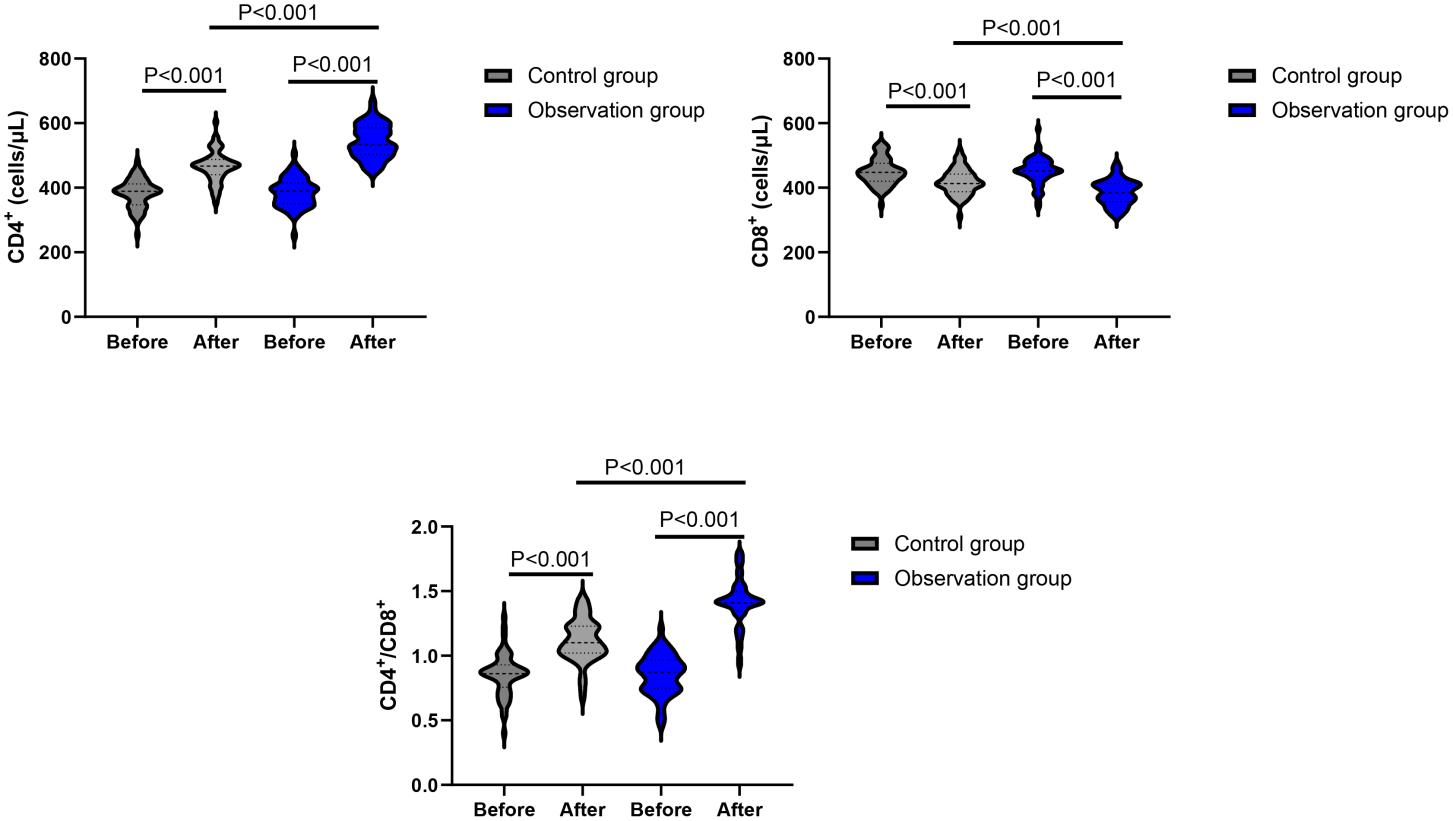
Immune-function indicators before and after treatment in the two groups. CD4+ and CD8+ T-cell counts are presented as mean ± standard deviation; the CD4+/CD8+ ratio is presented as median (interquartile range).

At baseline, no statistically significant between-group differences were observed in the CD4 + T-cell count, CD8 + T-cell count, or CD4+/CD8 + ratio (all *p* > 0.05). After treatment, the CD4 + T-cell count was significantly higher in the observation group than in the control group (542.34 ± 52.34 vs. 465.23 ± 48.56 cells/μL; mean difference, 77.10 cells/μL; 95% CI, 57.65 to 96.55). The CD8 + T-cell count was significantly lower in the observation group than in the control group (385.23 ± 38.45 vs. 418.34 ± 40.12 cells/μL; mean difference, −33.10 cells/μL; 95% CI, −48.24 to −17.96). Because the post-treatment CD4+/CD8 + ratio was not normally distributed in the observation group, it was summarized as median (interquartile range) and compared using the Mann–Whitney U test. The median CD4+/CD8 + ratio was significantly higher in the observation group than in the control group [1.41 (1.36–1.48) vs. 1.10 (1.02–1.23); U = 2,485]. All three comparisons remained statistically significant after Benjamini-Hochberg false discovery rate correction (all adjusted *p* < 0.001; Figure 5; Supplementary Table S1).

### ICU length of stay and mechanical ventilation duration

**Figure 6:**
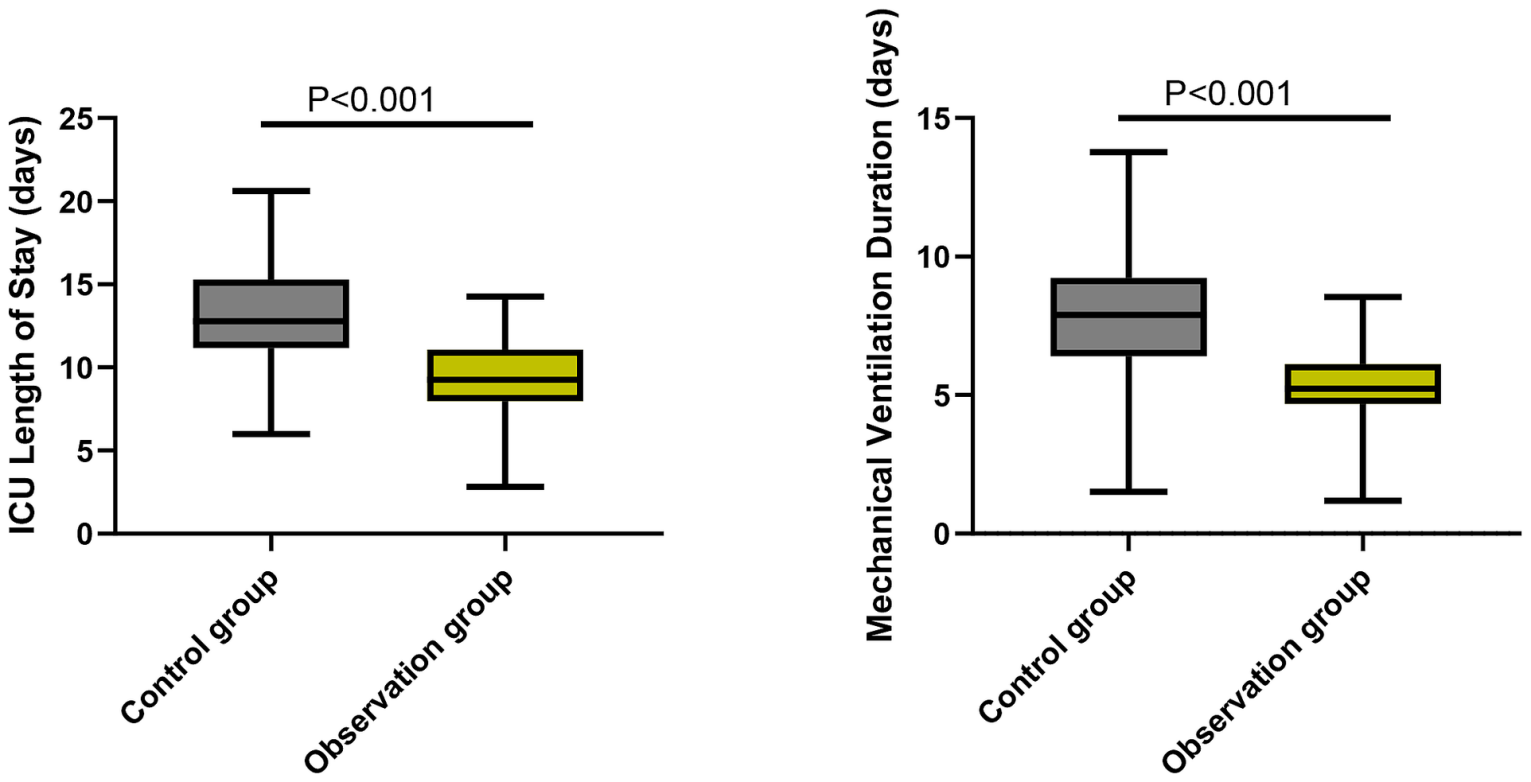
ICU length of stay and mechanical ventilation duration in the two groups. Box plots display the median, interquartile range, and range of the observed values. ICU length of stay was compared using an independent-samples *t*-test, and mechanical ventilation duration was compared using Welch’s *t*-test.

The ICU length-of-stay data were approximately normally distributed in both groups, and the homogeneity-of-variance assumption was satisfied. The mean ICU length of stay was significantly shorter in the observation group than in the control group (9.45 ± 2.34 vs. 12.78 ± 3.12 days; mean difference, −3.34 days; 95% CI, −4.40 to −2.28; *t* = −6.233; *p* < 0.001). Mechanical ventilation duration was also approximately normally distributed in both groups; however, the homogeneity-of-variance assumption was not met. Therefore, Welch’s *t*-test was used. The mean duration of mechanical ventilation was significantly shorter in the observation group than in the control group (5.23 ± 1.45 vs. 7.89 ± 2.35 days; mean difference, −2.65 days; 95% CI, −3.40 to −1.90; *t* = −6.991; *p* < 0.001) (Figure 6).

### Complications

ARDS, AKI, DIC, and MODS were recorded as individual non-fatal complications and were not mutually exclusive. The proportion of patients with at least one non-fatal complication was 9.4% (5/53) in the observation group and 24.5% (13/53) in the control group (risk ratio, 0.38; 95% CI, 0.15–1.00; *p* = 0.042) (Table 3).

**Table 3:** Comparison of non-fatal complications and 28-day all-cause mortality between the two groups.

Outcome	Observation group (*n* = 53)	Control group (*n* = 53)	χ^2^/risk ratio (95% CI)	*P*
ARDS	2 (3.8)	4 (7.5)	0.706	0.401
AKI	1 (1.9)	3 (5.7)	1.043	0.307
DIC	0 (0.0)	2 (3.8)	2.038	0.153
MODS	2 (3.8)	5 (9.4)	1.392	0.238
Any non-fatal complication	5 (9.4)	13 (24.5)	0.38 (0.15–1.00)	0.042
28-day all-cause mortality	2 (3.8)	6 (11.3)	0.33 (0.07–1.58)	0.143

### Twenty-eight-day mortality

Twenty-eight-day all-cause mortality occurred in 2 of 53 patients (3.8%) in the observation group and 6 of 53 patients (11.3%) in the control group. The difference was not statistically significant (risk ratio, 0.33; 95% CI, 0.07–1.58; *p* = 0.143) (Table 3).

## Discussion

SSS remains a critical challenge in intensive care medicine, with high morbidity and mortality rates despite advances in management strategies (14). The fundamental pathophysiology of septic shock involves an exaggerated inflammatory reaction induced by microbial invasion. This response drives widespread endothelial activation, disrupts microcirculatory function, causes coagulation disturbances, leads to inadequate tissue perfusion, and eventually culminates in multiple organ failure (15). Effective management requires not only prompt and appropriate antimicrobial therapy to eliminate the infectious source but also modulation of the exaggerated inflammatory cascade, restoration of coagulation homeostasis, improvement of tissue perfusion, and preservation of immune function.

Meropenem, a broad-spectrum carbapenem antibiotic, is widely recommended as the initial empirical treatment for severe sepsis and septic shock. Its strong bactericidal activity targets most clinically significant pathogens, including multidrug-resistant strains, making it highly effective in this setting (16). Its excellent tissue penetration and favorable safety profile make it an ideal choice for critically ill patients with suspicious or confirmed severe infections (17). However, antimicrobial therapy alone may be insufficient to control the uncontrolled inflammatory response that characterizes septic shock, as endotoxins released from bacterial lysis can further amplify the inflammatory cascade (18).

Ulinastatin, a urinary trypsin inhibitor, has been revealed to exert potent anti-inflammatory function by inhibiting the activity of various proteases, including trypsin, chymotrypsin, and elastase, thereby stabilizing lysosomal membranes and reducing the release of inflammatory mediators (19). Numerous studies have demonstrated that ulinastatin can attenuate the systemic inflammatory response, improve organ function, and diminish death rate in patients suffering from sepsis and septic shock (20). Additionally, ulinastatin has been shown to exert protective function on the coagulation system by inhibiting excessive activation of the coagulation cascade and preserving endothelial function (21). Furthermore, ulinastatin may improve microcirculatory perfusion and enhance lactate clearance by reducing endothelial dysfunction, stabilizing hemodynamics, and improving tissue oxygen delivery (22). The combination of meropenem and ulinastatin may represent a rational therapeutic strategy aimed at both the infectious etiology and the associated inflammatory pathophysiology of septic shock.

In this retrospective, non-randomized cohort, receipt of adjunctive ulinastatin was associated with a higher exploratory total effective rate. However, this composite outcome was retrospectively assessed, was not blinded, and had not been externally validated. It may therefore be affected by observer judgment, treatment-awareness bias, and residual confounding and should not be regarded as definitive evidence of treatment efficacy. Greater emphasis should be placed on objective patient-centered outcomes, particularly mortality. Although 28-day mortality was numerically lower in the observation group, the difference was not statistically significant and the confidence interval was wide because only eight deaths occurred.

Regarding inflammatory response, our study showed that the combination therapy resulted in significantly greater reductions in CRP, PCT, IL-6, IL-8, along with TNF-α levels relative to meropenem alone. CRP and PCT are widely used biomarkers for sepsis diagnosis and monitoring, with elevated levels correlating with disease severity and prognosis (23). The greater reduction in these markers in the observation group suggests that ulinastatin effectively suppressed the inflammatory cascade, potentially through repressing nuclear factor-κB (NF-κB) and subsequent cytokine production (24). IL-6, IL-8, along with TNF-α belong to crucial pro-inflammatory cytokines that play central roles in the pathogenesis of septic shock, contributing to endothelial activation, neutrophil recruitment, and tissue damage (25). The significant reduction of these cytokines in the observation group indicates that ulinastatin effectively attenuated the hyperinflammatory state, which may translate into improved organ function and reduced mortality. Consistently, Gao et al. demonstrated that the combination of ulinastatin and continuous blood purification markedly lowers inflammatory marker levels and reduces mortality among patients with sepsis, indicating its potential value in controlling sepsis-linked inflammation (26).

A particularly noteworthy finding of this study is the improvement in coagulation function in the observation group. Following treatment, Fib levels presented lower in the observation group versus the control group, while PT and APTT were shortened. These findings suggest an association between adjunctive ulinastatin use and improved coagulation parameters in this cohort. The underlying mechanism of sepsis-related coagulopathy involves the triggering of the coagulation cascade by inflammatory cytokines, which results in the depletion of coagulation factors, extension of PT and APTT, and reduction of Fib levels (27). Ulinastatin has been demonstrated to suppress the activation of coagulation factors as well as maintain endothelial integrity, thus halting the advancement of disseminated intravascular coagulation (DIC) (28). The improvement in coagulation parameters observed in our study suggests that ulinastatin may help restore hemostatic balance in SSS patients, potentially reducing the risk of thrombotic complications and organ dysfunction.

Lactate clearance is a clinically relevant indicator of tissue perfusion and is associated with outcomes in septic shock (29). The observation group had higher mean lactate clearance at 6 h (45.67 ± 8.92% vs. 32.45 ± 7.68%) and 24 h (68.34 ± 10.23% vs. 51.23 ± 9.45%). The observed association may reflect improved perfusion, differences in baseline management, or other measured and unmeasured factors; the present design cannot establish a causal mechanism.

Hemodynamic parameters improved in both groups, with the observation group presenting better restoration of HR, MAP, and CI. Septic shock is characterized by profound vasodilation, capillary leakage, and myocardial depression, leading to refractory hypotension and reduced tissue perfusion (30). The improved hemodynamic profile in the observation group may result from ulinastatin’s ability to stabilize endothelial function, reduce vascular permeability, and preserve myocardial function. By inhibiting the release of vasoactive mediators and protecting the endothelial glycocalyx, ulinastatin may contribute to the restoration of normal hemodynamic homeostasis (31).

Immune function is critically impaired in patients with septic shock, characterized by both excessive inflammation and simultaneous immunosuppression (32). CD4^+^ T helper cells are essential for coordinating adaptive immune responses, while CD8^+^ cytotoxic T lymphocytes are involved in eliminating pathogen-infected cells. The CD4^+^/CD8^+^ ratio belongs to a widely used indicator of immune system balance, with a reduced ratio indicating immune dysfunction (33). Our study demonstrated that the combination therapy significantly increased CD4^+^ T lymphocyte count and CD4^+^/CD8^+^ ratio, while decreasing CD8^+^ T lymphocyte count, suggesting that ulinastatin helps restore immune homeostasis. This immunomodulatory effect may be mediated through the inhibition of excessive apoptosis of lymphocytes, which is a hallmark of sepsis-induced immunosuppression (34). In agreement with our findings, Wang et al. reported that treatment with glutamine and ulinastatin helps alleviate septic shock primarily through the suppression of systemic inflammatory responses and enhancement of immune function (35).

The clinical outcomes in this study further support the benefits of the combination therapy. The ICU length of stay was shorter in the observation group, and the duration of mechanical ventilation was reduced. These findings indicate that the combination treatment not only improves physiological parameters but also translates into meaningful clinical benefits, including reduced resource utilization and potentially lower healthcare costs. The shorter duration of mechanical ventilation is particularly important given the associated risks of ventilator-associated pneumonia and other complications.

The observation group had a lower reported patient-level incidence of non-fatal complications. Mortality was analyzed separately and was numerically lower, but the study was underpowered for this endpoint and the difference was not statistically significant. Because individual ARDS, AKI, DIC, and MODS events may overlap within the same patient, event-specific counts should not be summed to derive the number of affected patients. Accordingly, mortality was reported separately and the composite non-fatal complication outcome counted each patient only once.

Several limitations should be recognized. First, this was a single-center retrospective, non-randomized study with a modest sample size; treatment selection, residual confounding, and selection bias limit causal inference and generalizability. Second, no *a priori* sample-size calculation was documented. Third, the total effective rate was a subjective composite outcome that was retrospectively assessed, was not blinded to treatment exposure, and had not undergone external validation; therefore, it was vulnerable to assessment bias and was treated only as an exploratory outcome. Fourth, the original study did not prospectively specify a single primary endpoint. Although 28-day mortality is the most clinically important objective outcome reported in this study, only eight deaths occurred, and the study was underpowered to detect a reliable between-group mortality difference. In addition, multivariable adjustment was not performed because of the limited sample size and low number of clinical events; therefore, residual confounding remains possible and the mortality and complication findings should be interpreted cautiously. Fifth, several standardized septic shock outcomes, including vasopressor duration, serial SOFA score trajectories, ventilator-free days, hospital mortality, and longer-term follow-up outcomes, were not consistently available in the retrospective source records and could not be analyzed. Future prospective studies should include these standardized patient-centered endpoints.

## Conclusion

In this single-center retrospective cohort, adjunctive ulinastatin use was associated with more favorable inflammatory, coagulation, lactate-clearance, hemodynamic, immune, and resource-use measures than meropenem-based standard care alone. The observational design, absence of randomization and blinding, subjective exploratory response outcome, multiple comparisons, and limited sample size preclude causal or “synergistic” conclusions. Prospective registration and adequately powered randomized trials are needed to determine whether ulinastatin improves patient-centered outcomes, particularly mortality.

## Data Availability

The original contributions presented in the study are included in the article/Supplementary material, further inquiries can be directed to the corresponding author.
