# Supplementary material for: Effects of meropenem plus ulinastatin on inflammatory response and complications in patients with severe septic shock
Source: Front Med (Lausanne). 2026 Jul 21;13:1860367. doi: 10.3389/fmed.2026.1860367 (PMC13433519; doi:10.3389/fmed.2026.1860367)
Supplement: Supplementary file 1 [file Table_1.docx]

**Supplementary Table S1 Effect estimates and 95% confidence intervals for post-treatment between-group comparisons**

| **Outcome** | **Control group** | **Observation group** | **Statistical test** | **Effect estimate** | **95% CI** | **FDR-adjusted P** |
| --- | --- | --- | --- | --- | --- | --- |
| CRP | 48.23 +/- 10.34 | 32.45 +/- 8.67 | t-test | MD: -15.78 | -19.46 to -12.10 | <0.001 |
| PCT | 2.67 +/- 0.56 | 1.56 +/- 0.45 | t-test | MD: -1.11 | -1.31 to -0.91 | <0.001 |
| IL-6 | 78.45 +/- 15.67 | 45.67 +/- 12.34 | t-test | MD: -32.78 | -38.22 to -27.34 | <0.001 |
| IL-8 | 62.34 +/- 12.56 | 38.45 +/- 10.23 | t-test | MD: -23.89 | -28.30 to -19.48 | <0.001 |
| TNF-alpha | 54.23 +/- 11.34 | 35.67 +/- 9.23 | t-test | MD: -18.56 | -22.54 to -14.58 | <0.001 |
| Fib | 2.12 +/- 0.42 | 1.78 +/- 0.34 | t-test | MD: -0.33 | -0.48 to -0.18 | <0.001 |
| PT | 14.23 +/- 1.78 | 12.45 +/- 1.56 | t-test | MD: -1.78 | -2.42 to -1.14 | <0.001 |
| APTT | 38.45 +/- 4.56 | 34.56 +/- 4.23 | t-test | MD: -3.89 | -5.59 to -2.19 | <0.001 |
| 6-h lactate clearance | 32.45 +/- 7.68 | 45.67 +/- 8.92 | t-test | MD: 13.22 | 10.01 to 16.43 | <0.001 |
| 24-h lactate clearance | 51.23 +/- 9.45 | 68.34 +/- 10.23 | t-test | MD: 17.10 | 13.31 to 20.89 | <0.001 |
| HR | 96.23 +/- 9.12 | 88.34 +/- 8.45 | t-test | MD: -7.88 | -11.27 to -4.49 | <0.001 |
| MAP | 71.34 (67.11-74.54) | 76.49 (73.37-83.73) | Mann-Whitney U test | U = 2239 | Not applicable | <0.001 |
| CI | 2.98 +/- 0.41 | 3.45 +/- 0.45 | t-test | MD: 0.48 | 0.31 to 0.65 | <0.001 |
| CD4+ T cells | 465.23 +/- 48.56 | 542.34 +/- 52.34 | t-test | MD: 77.10 | 57.65 to 96.55 | <0.001 |
| CD8+ T cells | 418.34 +/- 40.12 | 385.23 +/- 38.45 | t-test | MD: -33.10 | -48.24 to -17.96 | <0.001 |
| CD4+/CD8+ ratio | 1.10 (1.02-1.23) | 1.41 (1.36-1.48) | Mann-Whitney U test | U = 2485 | Not applicable | <0.001 |
| ICU length of stay | 12.78 +/- 3.12 | 9.45 +/- 2.34 | t-test | MD: -3.34 days | -4.40 to -2.28 | <0.001 |
| Mechanical ventilation duration | 7.89 +/- 2.35 | 5.23 +/- 1.45 | Welch's t-test | MD: -2.65 days | -3.40 to -1.90 | <0.001 |

Notes: Data are presented as mean +/- standard deviation or median (interquartile range), as appropriate. P values were adjusted using the Benjamini-Hochberg false discovery rate procedure within each outcome family. Abbreviations: 95% CI, 95% confidence interval; APTT, activated partial thromboplastin time; CD4+, cluster of differentiation 4-positive; CD8+, cluster of differentiation 8-positive; CI, cardiac index; CRP, C-reactive protein; FDR, false discovery rate; Fib, fibrinogen; HR, heart rate; ICU, intensive care unit; IL-6, interleukin-6; IL-8, interleukin-8; MAP, mean arterial pressure; MD, mean difference; PCT, procalcitonin; PT, prothrombin time; TNF-alpha, tumor necrosis factor-alpha.
